# “When Should I Tell?”: Perspectives on Disclosure to Their Children among Parents with Perinatally Acquired HIV

**DOI:** 10.3389/fpubh.2016.00140

**Published:** 2016-06-30

**Authors:** Cynthia D. Fair, Hannah Allen, Constance Trexler, Janet Osherow, Lawrence D’Angelo

**Affiliations:** ^1^Public Health Studies, Elon University, Elon, NC, USA; ^2^Elon University, Elon, NC, USA; ^3^Burgess Clinic, Children’s National Medical Center, Washington, DC, USA; ^4^Pediatric Infectious Disease Department, MedStar Georgetown University Hospital, Washington, DC, USA

**Keywords:** PHIV, disclosure, parenting, adolescent and young adult, children

## Abstract

Adolescents and young adults (AYA) with perinatally acquired HIV (PHIV) engage in developmentally expected behaviors, such as establishing relationships and having children. Previous research has focused on pregnancy management/outcomes of AYA with PHIV. However, little research has focused on the parenting experiences of this emerging cohort and on their views of disclosure to their offspring. This article examines data from a pilot study of five AYA parents with PHIV on disclosure to their child(ren) (*n* = 7, 6 HIV-negative). Disclosure of their own HIV status to their children is on the minds of parents with PHIV. However, few currently have children old enough to understand the parent’s diagnosis. Three parents indicated they would disclose their HIV status when their child was “old enough to understand” so that their child would be knowledgeable about HIV. One father also noted that he currently had more pressing parenting responsibilities beyond disclosure. When discussing their perspectives on disclosure, many referenced their personal stories indicating a link between their decision to disclose/not disclose to their child and their own disclosure narrative. One mother cited she did not plan to reveal her diagnosis to her son because he was uninfected, while another mother explained she did not want to worry her child. The mother of the only infected child “did not want to wait like my mother did” and planned to tell her son at an earlier age than when she learned of her own diagnosis. Clinical implications related to disclosure will be discussed and future areas of research identified.

Historically, children with perinatally acquired HIV (PHIV) were not expected to live to reach adolescence or adulthood (1). However, in the United States, about 22% of young adults with HIV, ages 13–24, have lived with the disease all their lives (2). Similar to many adolescents and young adults (AYA), this cohort is exploring romantic relationships, engaging in sexual activity, (3) and becoming parents (4). Previous research has focused on pregnancy management/outcomes of AYA with PHIV (5). However, little research has focused on the parenting experiences of this emerging cohort and on their views of disclosure to their offspring.

Although it is not possible to know how many AYA with PHIV currently have children, there are approximately 10,688 youth with PHIV living in the United States, and research has found that this population has high fertility desires/intentions, with over 80% expressing a desire to have a child in the future (6, 7). Childbearing motivations among AYAs with PHIV are strongly linked to experiences of parental loss due to illness, the wish to leave a legacy, and the desire to receive or offer unconditional love through the experience of parenting (8–10).

Experiencing parental loss or abandonment has a significant impact on any child, regardless of HIV status (11). HIV-affected children can face significant childhood trauma, including loss of one or both parents and a lack of domestic stability (12). The experience of living with a sick or dying parent due to AIDS can have a strong effect on the overall desire to have a child (10) and is also associated with high-risk sexual behavior during adolescence (13). The parenting experiences of AYA with PHIV may greatly color how they interact with their children and perceive their future and approach to parenting.

Adolescents and young adult parents with PHIV face many of the same challenges other young parents face, including financial worries and struggles with discipline (14). However, they also face HIV-specific issues, such as health concerns and the fear their child will experience HIV-related discrimination. Another unique feature of parenting with PHIV is whether and when to disclose their HIV status to their offspring, a topic largely unaddressed in the extant literature. Disclosure to a child about his or her parent with PHIV also, by default, discloses the HIV status of the child’s grandmother.

## Disclosure of Parental HIV Status to Children

Extensive literature has explored the effects of parental HIV disclosure to children. The review of related literature by Qiao et al. (15) found that parents living with HIV frequently considered the child’s age and cognitive level as well as perceived benefits of disclosure when deciding whether to share their HIV status. Additional factors included concerns over stigma and possible discrimination. While the literature on the effects of parental HIV disclosure on child functioning is mixed, the preponderance of evidence points to positive long-term outcomes for children, especially among children who were informed at younger ages (16). For example, Tompkins (17) reported that children who were informed of their mother’s HIV status felt better prepared for the future and expressed pride in their ability to reduce their mother’s stress. Research has also found psychological benefits to parents following HIV disclosure within a sample of HIV-affected families in rural South Africa (18).

To date, limited literature has explored disclosure to children of parents with PHIV. In a small qualitative study, Evangeli et al. (19) found that four out of seven participants discussed disclosing their HIV status to their children frequently reflecting upon their own disclosure experiences. However, it is unclear whether they were parents at the time of the study. The purpose of this pilot study was to explore views of disclosure to their offspring among a sample of parents with PHIV.

## Pilot Study

### Participants

A purposive sample of five AYA with PHIV (four females) who had children were recruited from an urban tertiary-care facility in the southeast United States (mean age = 23.4 years, range 23–24). All identified as heterosexual and African-American. Mean number of children was 1.4 (*n* = 7, range 1–3; mean age = 2.4 years, range 3 months–4 years), and one child was HIV-positive. Six patients met study eligibility and five participated. One patient was unable to participate due to scheduling conflicts.

### Procedure

A staff member from the clinic introduced the study to eligible patients. Audio-recorded, semi-structured, face-to-face interviews were conducted by a trained interviewer (Hannah Allen) and later transcribed. Participants were provided a $50 gift card in compensation for their participation. The study was approved by the hospital’s IRB.

Sample questions included:

What thoughts do you have about disclosing your diagnosis to your child?

If you’re going to tell your child, how are you going to tell her/him?

### Data Analysis

Transcribed interviews were entered into Atlas.ti 7.0, a qualitative data analysis software program (20). The authors used a grounded theory approach to analyze the data, which employs an inductive strategy designed to identify emergent themes (21, 22). Cynthia D. Fair and Hannah Allen independently read the interview transcripts in their entirety, and following the traditions of the grounded theory method, analysis began with a process of open coding. The readers met frequently to discuss identified themes and to come to consensus on the coding.

## Results

At the time of the study, all of the participants had disclosed to their current significant other or the other parent of their child. However, two participants had not disclosed their HIV status to their partners at the time their children were born. All were in serodiscordant relationships. One of the participants had disclosed her status publicly and considered “the world” to know her status. None of the parents had told any friends about their illness, yet, disclosure to offspring was on their minds. However, few currently had children old enough to understand their parents’ diagnosis. Findings indicated that their own disclosure experience, when and how they were told about their own HIV status, heavily influenced whether or not they planned to disclose to their child.

*Yes, I’m definitely going to disclose it to him*.

Three participants planned to one day tell their child(ren) about their HIV status, including the mother of the HIV-infected child. They stressed the importance of timing, indicating that a child should not be told at a very young age, but it was also important for the child not to find out from someone else. Participants noted that their children were smart and, as a result, would be able to understand their parent’s HIV diagnosis.

A 24-year-old mother, currently in school, said she wanted to tell her daughter similar to how she had been told of her own diagnosis at the hospital through a “developmental type of a program” where, she said, “I remember drawing pictures of HIV, the blood cells…. I remember learning through pictures and them explaining it to me the reason why I was coming here.” She explained that she accidentally told a friend she had HIV when she was younger and was ostracized as a result. “You don’t understand what it is, and you don’t understand. When you hear a certain term, you think negative. I think that’s what happened in that situation [with my friend].” She explained it was important that her daughter understand the risk of rejection. “I feel like I should tell her when she’s in school and beginning to learn about it” because she fears her daughter might say “Mommy has it.” She also noted it was important for her daughter to have accurate information about HIV, “When you’re learning about it in school, they don’t actually explain it … For example, born with HIV and then being acquired with HIV like there’s sex and drugs and anything. It’s clearly different.”

The only father in the study indicated his son would learn of his father’s HIV status at some point in the future stating, “I have to tell him one day.” However, his son was only 3 years old and other parenting demands took priority. When asked to speak about how he might disclose to his son, he explained that he had not disclosed his HIV diagnosis to anyone. Someone else informed his son’s mother about his HIV status. He expressed remorse over his reluctance to disclose prior to engaging in sexual activity but stated, “I didn’t know how to tell her, I don’t know how to go about it.”

The participant whose child had HIV was a 24-year-old employed mother who lived with her brother and son. She recognized that her situation was different because her child would have to learn of his HIV status. She indicated she had given disclosure a great deal of reflection stating, “I’ve thought about it [how to disclose to him] a lot.” Referencing her own disclosure, she said, “I don’t want it to be a wait, like how my mom did … I don’t want it to be like that with my son, I want him to be aware ahead of time so he can know.”

*I have no reason to*.

Two parents indicated they would not share their HIV status with their child. One 23-year- old participant received dialysis and had a difficult pregnancy. She referenced her own experience of finding out her status stating that she “overheard [her mom and others] talking, so [she] thought it was a good thing, because … Christmas was coming up, so I thought it was a surprise.” She explained that her mom had planned on waiting longer to tell her because “I was 8 years old. She [her mom] wasn’t going to tell me at that age, because I didn’t understand what it was.” Her disclosure experience was negative and, as a result, she did not want to tell her son about her own status. She explained that he probably did not have HIV, based on the tests so far and that “most likely it’s [the third test] going to be negative, so if he don’t have it, I don’t plan on telling him” that she has HIV.

The other parent who planned not to disclose her status to her children was a 24-year-old who lived in transient housing, was unemployed, and had lost custody of her three children. She was also the participant who claimed “the world” knew her status because she was very open about it. However, she said that she did not want to disclose to her children “because I don’t want my kids to be worried about me.”

## Clinical Implications

The purpose of this study was to explore the disclosure perspectives of parents with PHIV and to draw upon the extant literature as well as the clinical experiences of the authors to highlight potential issues related to this emerging phenomena. Empirically based conclusions cannot be made from the study findings, yet they can serve as a springboard for future research and clinical considerations.

Clinical implications of disclosure to children of parents with PHIV depend upon both child- and parent-related factors. In general, timing of the disclosure should be based on the child’s cognitive development and ability to understand the concept of illness (23). Furthermore, if the child is also infected then he/she will need to be told sooner as previous research indicates that children who are aware of their HIV diagnosis are more likely to be fuller partners in their care (24). Many pediatric HIV clinics have structured disclosure programs that provide a developmentally appropriate, and collaborative process involving both the caregiver and health-care provider to disclose a child’s HIV status to him or her. Findings from the exploratory study highlight the importance of planned and intentional disclosure to children.

Resources addressing disclosure of HIV status to children are mostly geared to disclosure to a child with PHIV regarding his/her diagnosis and not a parent’s personal diagnosis to the child (25). Therefore, if the child is uninfected there may be less support from care providers related to disclosure. Additionally, resources diminish as an adolescent ages into adult care. The social support and services that were once prevalent in the pediatric/adolescent clinics are stretched thin in the adult care setting due to the increased number of patients and different funding structures. Options for disclosure assistance and counseling for the adult with PHIV already in adult care are limited. Our findings suggested that those parents with PHIV who plan to share their HIV status with their child wish to wait until their child is older. Parents will likely be receiving care in an adult infectious diseases clinic at that time where disclosure is usually seen within the context of newly diagnosed patients or a sexual partner. HIV providers in adult clinics who are treating parents with PHIV may have limited experience with or resources for disclosure to a child. Parents may need support as they disclose to their child.

The World Health Organization and other organizations have readily available resources, which provide guidance on disclosure to children with HIV (23, 26). Cavolo et al. (25) note the cornerstones of disclosure to children with PHIV include developmentally appropriate and truthful explanations of the illness, validation of the child’s concerns about the illness, clarification of any misconceptions, and ongoing support. These principles could also be applied when a parent discloses his/her HIV diagnosis to their child.

In addition to the child’s developmental and HIV status, the health of the parent and his/her disclosure history should also be taken into account (23, 25). If the parent is asymptomatic, then the parent can afford to wait to give the child insight into the parent’s health. However, delayed disclosure can result in accidental disclosure, which may lead to feelings of distrust as found in several of the disclosure narratives shared by the participants (27). Previous research indicates that, over time, children and adolescents adjust well to learning their parent’s HIV status (28).

Consideration should also be given to the parent’s acceptance of his or her own illness and history of disclosing to others as evidenced by the young man who felt he did know how to disclose his HIV status to the mother of his child. Parents who had negative disclosure experiences, either when they learned of their own diagnosis or when they told others, may need additional support when and if they choose to tell their child, since they have not had access to models of supportive disclosure processes. Those parents with PHIV who have had limited opportunities to disclose their status to friends, family, and/or sexual partners, will likely feel less comfortable talking about their illness with others, including their offspring. Furthermore, none of the participants had disclosed to their friends highlighting the fact that, despite reduced levels of HIV-related stigma, disclosure is still difficult. Health-care providers can help young adults with HIV practice disclosure through role play, helping them think through possible questions their child might ask following disclosure (29).

## Intergenerational Effects of Disclosure

Parental disclosure to offspring among parents with PHIV has implications beyond their own HIV diagnosis as it also necessarily discloses a grandmother’s HIV status. There are several levels of disclosure for the child to ultimately understand. The most immediate disclosure is between the parent and child, followed by the grandparent and child. A child must first learn that his/her parent has HIV, then learn that his/her parent has been infected with HIV since birth. For a young child, the story can be simple and factual, but without extensive discussions of how or why. However, older children and adolescents may have additional questions due to HIV education or their own misconceptions about HIV. They may be subject to external influences possibly leading to self-stigma with fear of discrimination by others. Indeed, Woodring et al. (30) found that adolescents who learned of their parent’s HIV status were concerned about who they could talk to for fear of rejection.

Adolescents will likely realize the implication of the parent being infected since birth means a grandparent is or was also infected with HIV. The questions could quickly shift from “how did my parent become infected” to “how did my grandmother get this disease” or even “why did my grandmother give this to my parent?” This new knowledge could bring to the forefront conversations which have been taboo for the past two generations. A culture of secrecy and silence can lead to feelings of insecurity and fears of loss for the child. Intergenerational parenting styles are not always carried through to the next generation (11). With strong relationships and social support from medical and social service providers, maladaptive communication patterns can be changed opening the way for family secrets to be discussed promoting intergenerational respect.

Findings from the pilot study confirmed previous research, which found that youth with PHIV reflected upon their own disclosure story when considering disclosure to future children (19). This underscores the importance of facilitating positive disclosure processes with those newly diagnosed with HIV. Based on these findings and the other clinical implications outlined above, it can be determined that disclosure counseling needs to be a regular part of psychosocial care for those diagnosed with HIV. In order for those services to be provided, high-quality research is needed on the process of disclosure within this unique and emerging population.

To date, many pediatric and adolescent HIV-care providers are well versed in the psychosocial considerations surrounding disclosure to children. However, adult providers will ultimately care for the vast majority of young adult parents with PHIV and, therefore, will be the point of contact for disclosure counseling for their children. Adult providers may need additional training related to disclosure support in order to promote positive disclosure experiences between parents with HIV and their children.

Disclosure to children from the perspective of parents living with PHIV is understudied. Our exploratory findings indicated disclosure is complex and linked with the parent’s own disclosure experience. As more AYA with PHIV mature into adulthood, disclosure to their children will become more commonplace, especially in regions of the world with high rates of maternal HIV infection, such as sub-Saharan Africa.

## Conclusion

It is unknown if disclosure outcomes of parents with PHIV will parallel previously published research on disclosure outcomes of parents with behaviorally acquired HIV. Longitudinal prospective studies focused on the natural history of disclosure across the lifespan of AYA with PHIV are necessary to identify ways to better support and promote optimal functioning.

## Author Contributions

CF was lead author on this project. She helped analyze data, write, edit, and coordinate correspondence with other coauthors. HA conducted all the interviews and analyzed the data. She also helped to write and edit the paper. CT wrote the IRB and made significant contributions to the body of the paper. JO contributed to the writing of the paper and offered clinical expertise. LD was PI on the study and made significant contributions to the body of the paper.

## Conflict of Interest Statement

The authors declare that the research was conducted in the absence of any commercial or financial relationships that could be construed as a potential conflict of interest.
